# No association between telomere length-related loci and number of cutaneous nevi

**DOI:** 10.18632/oncotarget.12021

**Published:** 2016-09-14

**Authors:** Xin Li, Geyu Liang, Mengmeng Du, Immaculata De Vivo, Hongmei Nan

**Affiliations:** ^1^ Department of Epidemiology, Harvard T.H. Chan School of Public Health, Boston, MA, USA; ^2^ Key Laboratory of Environmental Medicine Engineering, Ministry of Education, School of Public Health, Southeast University, Nanjing, Jiangsu, China; ^3^ Department of Epidemiology and Biostatistics, Memorial Sloan Kettering Cancer Center, New York, NY, USA; ^4^ Channing Division of Network Medicine, Brigham and Women's Hospital and Harvard Medical School, Boston, MA, USA; ^5^ Department of Epidemiology, Richard M. Fairbanks School of Public Health, Indiana University, and Indiana University Melvin and Bren Simon Cancer Center, Indianapolis, IN, USA

**Keywords:** telomere length-related loci, melanoma, nevus count, genome-wide association study, genetic score

## Abstract

**Background:**

Longer telomeres have been associated both with increased melanoma risk and increased nevus counts. Nevus count is one of the strongest risk factors for melanoma. Recent data showed that a genetic score derived by telomere length-related single nucleotide polymorphisms (SNPs) was strongly associated with melanoma risk; however, the relationships between these SNPs and number of cutaneous nevi have not been investigated.

**Methods:**

We evaluated the associations between telomere length-related SNPs reported by previous genome-wide association study (GWAS) and nevus counts among 15,955 participants of European Ancestry in the Nurses' Health Study and Health Professionals Follow-up Study.

**Results:**

None of the SNPs was associated with nevus counts, nor was the genetic score combining the dosage of alleles related to increased telomere length.

**Conclusions:**

The telomere length-related SNPs identified by published GWAS do not appear to play an important role in nevus formation. Genetic determinants of telomere length reported by GWAS do not explain the observed epidemiologic association between telomere length and nevus counts.

## INTRODUCTION

Telomeres are long hexameric (TTAGGG)n repeats at the ends of linear eukaryotic chromosomes that shorten after each round of DNA replication. They play a critical role in maintaining genomic stability [1]. Nevi occur when melanocytes cluster, and the number of melanocytic nevi in sun-exposed sites is a strong risk factor for cutaneous melanoma [2]. Han and colleagues found that shorter telomeres were associated with both a lower risk of melanoma and a decreased nevus count [3, 4]. In addition, positive associations between telomere length (TL) and nevi number and size have been reported previously [5]. It has been hypothesized that these phenotypes share common genetic components. In a recent study by Iles *et al.* [6]. The authors discovered a highly statistically significant association between a genetic score derived by TL-related SNPs and risk of melanoma (*P*-value = 8.92 × 10^−9^). However, it is unclear whether these genetic variants also influence the number of cutaneous nevi. We therefore evaluated the relationship between TL-related single nucleotide polymorphisms (SNPs) and nevus count.

## RESULTS

We did not observe significant associations between any of the seven TL-related SNPs and nevus count (Table 1). Mean values and ranges of the genetic scores combining all 7 independent SNPs were similar among the Illumina, Affy, and Omni datasets. We found that genetic scores were not significantly associated with nevus count in our data. The results for simple count score (beta = 0.004, *p*-value = 0.33) and weighted score (beta = 0.005, *p*-value = 0.42) were similar (Table 2). We calculated the power using the pwr package for multiple linear regression in R; our data had 99% power at two-sided alpha of 0.05 to detect an effect size beta of 0.004.

**Table 1:** Results for each telomere length-related SNP, including their associations with melanoma risk and nevus count

SNP	Chr	Position	Related gene	Minor allele	MAF in telomere GWAS [13]	MAF in our data	Average imputation R-square^1^	Melanoma beta^2^	Melanoma *P* value^2^	Nevus count beta	Nevus count *P* value
rs10936599	3	169492101	*TERC*	T	0.252	0.241	0.993	−0.079	0.0003	−0.020	0.48
rs2736100	5	1286516	*TERT*	C	0.486	0.506	0.988	0.078	0.02	−0.043	0.08
rs7675998	4	164007820	*NAF1*	A	0.217	0.222	0.988	−0.063	0.03	−0.036	0.22
rs9420907	10	105676465	*OBFC1*	C	0.135	0.142	1.000	0.083	0.001	0.051	0.14
rs8105767	19	22215441	*ZNF208*	G	0.291	0.290	0.996	0.028	0.16	0.025	0.34
rs755017	20	62421622	*RTEL1*	G	0.131	0.123	0.952	0.026	0.35	0.004	0.92
rs11125529	2	54475866	*ACYP2*	A	0.142	0.134	0.999	−0.004	0.86	0.020	0.56

1 Average imputation R square quality metric of Illumina, Affy, and Omni datasets;

2 Information on associations between SNPs and risk of melanoma was obtained from paper by Iles et al. [6].

**Table 2:** Associations between genetic scores (GSs) of telomere length-related SNPs and nevus count^1^

	Illumina			Affy			Omni			Meta-analysis^2^		
	Beta	SE	*P*-value	Beta	SE	*P*-value	Beta	SE	*P*-value	Beta	*P*-value	P Heterogeneity
**Simple count GS**	0.003	0.008	0.67	0.009	0.007	0.21	−0.001	0.008	0.90	0.004	0.33	0.66
**Weighted GS**	0.004	0.008	0.64	0.009	0.007	0.21	−0.004	0.009	0.64	0.005	0.42	0.50

1 Linear regression models were used to assess the relationship between genetic scores (rescaled) and nevus count, adjusting for age, gender, and the top three principle components;

2 Analyses were first conducted within each of the platform-specific genetic datasets. We used fixed-effect meta-analysis to obtain a combined estimate.

## DISCUSSION

Despite epidemiologic evidence of a link between TL and nevus count, and the strong association between TL-related SNPs and melanoma, we did not detect an association between TL GWAS SNPs and number of nevi. This is consistent with findings of some experimental studies that telomere attrition may not be involved in the initial formation of melanocytic nevi, because telomere length in nevi did not differ significantly from surrounding tissue and nevi generally lack expression of p53 and p21, markers of telomere-induced senescence [7]. In addition, both these phenotypes have long been recognized as the result of combined genetic and environmental risk factors. Thus, the reported association might be largely due to shared environmental risk factors (such as UV exposure) or regulations beyond the gene level. Furthermore, it is possible that the selected SNPs do not cover those genes involved in both telomere length and nevus count, as our knowledge of TL-related loci remains incomplete. In summary, we did not find evidence of associations between the selected TL-related alleles and nevus count. This suggests that the effect of telomere genetic score on melanoma risk may not be mediated through nevus counts. Future work is needed to screen more novel TL-related genetic variants, and investigate the association between these phenotypes at the level of other “-omics”.

## MATERIALS AND METHODS

Eighteen case-control studies nested within the Nurses' Health Study (NHS) and the Health Professionals Follow-up Study (HPFS) were combined into three compiled datasets based on their genotype platforms: Affymetrix (Affy), Illumina HumanHap series (Illumina), or Illumina Omni Express (Omni) (Supplementary Tables S1 and S2). Detailed descriptions on study population, genotyping procedure, quality control and imputation are provided in Supplementary Materials.

In total, 15,955 participants of European ancestry were included in the current study. In both the NHS and HPFS, participants were asked to provide information on melanocytic nevus count (larger than 3 mm in diameter) on arms by choosing from the following categories: 1 = none, 2 = 1–2, 3 = 3–5, 4 = 6–9, 5 = 10–14, 6 = 15–20, and 7 = 21+. Previous studies have evaluated the validity of self-reported information on nevus counts. The majority of studies on nevus counts have shown substantial agreement between nevus self-counts and dermatologist counts [8, 9]. The Spearman's correlation coefficient was 0.91 between the two measurements [10]. In our cohorts, the ordinal variable of nevus count is a highly significant predictor of melanoma risk [11]. A genome-wide association study on nevi number using our genetic datasets has revealed biologically plausible susceptibility loci for nevus count and melanoma risk, and the same loci were replicated by other independent studies. In the current study, we used number of nevi on the arms as proxy for the total baby nevus counts, which has been proven to be acceptable [12].

We obtained seven independent TL-related SNPs from the largest genome-wide association study (GWAS) on TL [13]. The same SNPs were examined by Iles *et al.* [6] for the association with melanoma risk. For each participant, we summed the dosage of alleles related to increase in TL of the seven SNPs to obtain the simple count genetic score. We also constructed a weighted score by multiplying the dosage of effect alleles by the corresponding regression coefficients in the original GWAS paper [13] and then summing the products. Both the simple count score and the weighted score were rescaled to a mean of 14 alleles (2 alleles * 7 SNPs) before testing their associations with nevus count to make results comparable. We presented formula and summary statistics of the genetic scores in Supplementary Table S3.

We regressed nevus counts (0 = none, 1.5 = 1–2, 4 = 3–5, 7.5 = 6–9, 10 = 10+) on the dosage of each SNP and the genetic scores, respectively, adjusted for age, sex, and the top three principle components (PCs). All the analyses were first conducted within each of the platform-specific datasets, and then combined by inverse-variance-weighted meta-analysis if results were not significantly different. ProbABEL package and R-3.0.2 were used to perform these tests. We considered 2-sided *P* values less than 0.05 to be statistically significant.

## SUPPLEMENTARY MATERIALS


