# Supplementary material for: No association between telomere length-related loci and number of cutaneous nevi
Source: Oncotarget. 2016 Sep 14;7(50):82396–9. doi: 10.18632/oncotarget.12021 (PMC5347699; doi:10.18632/oncotarget.12021)
Supplement: Supplementary file 1 [file oncotarget-07-82396-s001.pdf]

No association between telomere length-related loci and number of cutaneous nevi

Supplementary Materials

STUDY POPULATION

Nurses' health study (NHS)

The NHS is a prospective cohort study established in 1976 with 121,700 female U.S. registered nurses, who were then 30–55 years old. All of them completed and returned a mailed self-administered questionnaire about their medical histories and baseline lifestyle. In 1989 and 1990, a total of 32,826 women provided blood samples. Information regarding medical history, lifestyle, and disease diagnoses was updated every two years with a follow-up rate of 90%.

Health professionals follow-up study (HPFS)

The HPFS began in 1986 with 51,529 U.S. male health professionals who were 40–75 years old at initial recruitment. They all answered a detailed mailed questionnaire at the inception of the study. Disease- and health-related information was obtained and updated through biennial questionnaires. Between 1993 and 1994, 18,159 of these men provided a blood sample. The average follow-up rate for this cohort over 10 years is greater than 90%.

Basic information on the 18 nested case-control studies

Genotyping, quality control, and imputation

Genotyping

There were 18 GWAS datasets from the NHS and HPFS with cleaned genotype data available. We combined these datasets into three complied datasets based on their genotype platform type: Affymetrix (Affy), Illumina HumanHap series (Illumina), or Illumina Omni Express (Omni). The Affymetrix dataset was comprised of data on the Affy 6.0 platform (NHS-type 2 diabetes, NHS-coronary heart disease, HPFS-type 2 diabetes, HPFS-coronary heart disease). The Illumia HumanHap dataset was comprised of several platforms: Illumina 550K (NHS-breast cancer, NHS-pancreas cancer, HPFS-pancreas cancer), Illumina 610Q (NHS-kidney stone, HPFS-kidney stone, HPFS-prostate cancer) and Illumina 660 (NHS-glaucoma, HPFS-glaucoma). The Illumina Omni Express dataset contained only studies genotyped on the Omni Express platform (NHS-endometrial cancer, NHS-colon cancer, NHS-mammographic density, NHS-gout, HPFS-colon, HPFS-gout). Detailed method about the pooled imputed data in this combined dataset is described in Lindström, et al. submitted to Bioinformatics (copy is provided for reviewers' review).

Quality control (QC)

We combined the individual datasets that were genotyped on the same platform, removing any SNPs that were not in all studies and with a missing call rate > 5%, and flipping strands where appropriate to create a final compiled dataset. This resulted in 668,283 SNPs in the Affymetrix dataset, 459,999 SNPs in the Illumina HumanHap dataset, and 565,810 SNPs in the Illumina Omni Express dataset. Analyses were restricted to subjects with self-reported European ancestry. Genetic principal components were calculated using sets of independent SNPs (12,000-33,000 SNPs depending on platform). Subjects who did not cluster with other self-identified Europeans based on the top five principal components were also excluded.

We then ran a pairwise identity by descent (IBD) analysis for each combined dataset to detect duplicate and related individuals based on resulting Z scores. If $0 <= Z0 <= 0.1$ and $0 <= Z1 <= 0.1$ and $0.9 <= Z2 <= 1.1$ then a pair was flagged as being identical twins or duplicates. Pairs were considered full siblings if $0.17 <= Z0 <= 0.33$ and $0.4 <= Z1 <= 0.6$ and $0.17 <= Z2 <= 0.33$. Half siblings or avunculars were defined as having $0.4 <= Z1 <= 0.6$ and $0 <= Z2 <= 0.1$. Some of the duplicates flagged in this step were expected, having been genotyped in multiple datasets and hence having the same cohort IDs. In this case, one of each pair was randomly chosen for removal from the dataset. Instances where pairs were flagged as unexpected duplicates with the different cohort IDs, but pairwise genotype concordance rate > 0.999, resulted in removal of both individuals from the pair.

Supplementary Table S1: Basic information on the 18 GWAS sets from NHS and HPFS

Study	Sample size * (Genotyped)	Genotyping platform	Combined dataset
Postmenopausal invasive breast cancer case-control study nested within the NHS (NHS-BrCa)	1145 cases, 1142 controls	Illumina 550k	Illumina
Type 2 diabetes case-control study nested within the NHS (NHS-T2D)	1532 cases, 1754 controls	Affy 6.0	Affy
Coronary heart disease case-control study nested within the NHS (NHS-CHD)	342 cases, 804 controls	Affy 6.0	Affy
Kidney stone case-control study nested within the NHS (NHS-KS)	328 cases, 166 controls	Illumina 610Q	Illumina
Pancreas cancer case-control study nested within the NHS (NHS-Pancreas)	82 cases, 84 controls	Illumina 550k	Illumina
Glaucoma case-control study nested within the NHS (NHS-Glaucoma)	313 cases, 497 controls	Illumina 660	Illumina
Endometrial cancer case-control study nested within the NHS (NHS-Endometrial)	396 cases, 348 controls	Omni Express	Omni
Colon cancer case-control study nested within the NHS (NHS-Colon)	394 cases, 774 controls	Omni Express	Omni
Mammographic density study nested within the NHS (NHS-Mammographic density)	153 cases, 641 controls	Omni Express	Omni
Gout case-control study nested within the NHS (NHS-Gout)	319 cases, 392 controls	Omni Express	Omni
Type 2 diabetes case-control study nested within the HPFS (HPFS-T2D)	1189 cases, 1298 controls	Affy 6.0	Affy
Coronary heart disease case-control study nested within the HPFS (HPFS-CHD)	435 cases, 878 controls	Affy 6.0	Affy
Kidney stone case-control study nested within the HPFS (HPFS-KS)	315 cases, 238 controls	Illumina 610Q	Illumina
Pancreas cancer case-control study nested within the HPFS (HPFS-Pancreas)	54 cases, 52 controls	Illumina 550k	Illumina
Advanced prostate cancer case-control study nested within the HPFS (HPFS-AdvPrCa)	218 cases, 205 controls	Illumina 610Q	Illumina
Glaucoma case-control study nested within the HPFS (HPFS-Glaucoma)	178 cases, 299 controls	Illumina 660	Illumina
Colon cancer case-control study nested within the HPFS (HPFS-Colon)	229 cases, 230 controls	Omni Express	Omni
Gout case-control study nested within the HPFS (HPFS-Gout)	717 cases, 699 controls	Omni Express	Omni

* These are number of participants who have been genotyped in each of the studies before imputation, quality control, and further exclusion. Cases refer to the cases of disease in the original nested case-control study.

Related individuals (full sibs, half sibs/avunculars) were not removed from the final datasets. In the Affymetrix dataset, 167 individuals were removed because they were duplicates or were flagged for removal from secondary genotype data cleaning, leaving a total of 8065 individuals. Of the 6894 individuals originally in the Illumina dataset, 107 were removed because they were duplicates or flagged for removal in the genotyping step, leaving 6787 IDs. In addition, 8 pairs of individuals were flagged as related. In the Omni express dataset, there were 5956 individuals at the start, with 39 IDs to remove leaving 5917 IDs and 5 pairs of related IDs.

After removing duplicate IDs and flagging related pairs of IDs, we used EIGENSTRAT [1] to run PCA analysis on each compiled dataset, removing one member from each flagged pair of related individuals. For Affymetrix and Illumina HumanHap, we used approximately 12,000 SNPs that were filtered to ensure low pairwise LD. For the OmniExpress dataset we used approximately 33,000 SNPs that were similarly filtered.

Supplementary Table S2: Summary of markers in combined datasets

Platform	# of markers in cleaned and merged datasets	Total # of 1000G imputed markers	# of 1000G imputed markers with MAF > 1%	# of 1000G imputed markers with MAF > 1% and imputation R^2 > 0.3
Affymetrix (Affy)	668,283	31,326,389	9,783,513	9,783,513
Illumina (Illumina)	459,999	31,326,389	9,807,739	8,991,321
Omni Express (Omni)	565,810	31,326,389	9,771,868	9,148,255

Supplementary Table S3: Formula and summary statistics of simple count and weighted genetic scores

		Illumina	Affy	Omni
Simple count genetic score	Original Mean(range)	5.46 (0.004, 10.04)	5.47 (0.04, 10.98)	5.48 (1.01, 11.00)
	Formula	$2.566(\sum_{i=1}^{7} SNP_i)$	$2.558(\sum_{i=1}^{7} SNP_i)$	$2.554(\sum_{i=1}^{7} SNP_i)$
	Rescaled Mean(range)	14 (0.01, 25.76)	14 (0.09, 28.09)	14 (2.57, 28.09)
Weighted genetic scoreMean(range)	Original Mean(range)	0.42 (0.0003, 0.75)	0.42 (0.003, 0.80)	0.42 (0.05, 0.80)
	Formula	$33.413(\sum_{i=1}^{7} SNP_i)$	$33.413(\sum_{i=1}^{7} SNP_i)$	$33.254(\sum_{i=1}^{7} SNP_i)$
	Rescaled Mean(range)	14 (0.01, 24.93)	14 (0.10, 26.73)	14 (1.63, 26.67)

We plotted the top eigenvectors using R and examined the plots for outliers.

Finally, as a quality control check, we ran logistic regression analyses using each individual study's controls as "cases" and the rest of the studies controls as "controls". We then ran regressions with each of the other study controls as "cases" versus all of the rest of the controls. We looked for p values of genome-wide significance ($p < 10^{-8}$) and examined QQ plots to determine if any SNPs were flagged as significant where no SNPs should have been significant. In the Affymetrix dataset 100 SNPs were flagged and removed. In the Illumina HumanHap dataset, 8 SNPs had $p < 10^{-8}$ in any of the QC regressions and were removed. No SNPs in the Illumina Omni Express dataset had p values $< 10^{-8}$, hence no additional SNPs needed to be removed. After the datasets were combined and appropriate SNP and ID filters applied, the complied datasets were imputed.

Imputation

After the datasets were combined and appropriate quality control procedures applied, the complied datasets were imputed using the 1000 Genomes Project ALL Phase I Integrated Release Version 3 Haplotypes excluding monomorphic and singleton sites (2010-11 data freeze, 2012-03-14 haplotypes) as reference panel. SNP genotypes were imputed in three steps. First, genotypes on each chromosome were split into chunks to facilitate windowed imputation in parallel using ChunkChromosome (http://genome.sph.umich.edu/wiki/ChunkChromosome, v. 2011-08-05). Then each chunk of chromosome was phased using MACH (v. 1.0.18.c) [2]. In the final step, Minimac (v. 2012-08-15) [3] was used to impute the phased genotypes to approximately 31 million markers in the 1000 Genomes Project. The number of genotyped SNPs passed quality control procedure and that of imputed SNPs

with minor allele frequency (MAF) > 1% and imputation R2 > 0.3 in each platform are presented in Supplementary Table S2.

REFERENCES

1. Price AL, Patterson NJ, Plenge RM, Weinblatt ME, Shadick NA and Reich D. Principal components analysis corrects for stratification in genome-wide association studies. Nature genetics. 2006; 38:904–909.
2. Li Y, Willer CJ, Ding J, Scheet P and Abecasis GR. MaCH: using sequence and genotype data to estimate haplotypes and unobserved genotypes. Genetic epidemiology. 2010; 34:816–834.
3. Howie B, Fuchsberger C, Stephens M, Marchini J and Abecasis GR. Fast and accurate genotype imputation in genome-wide association studies through pre-phasing. Nature genetics. 2012; 44:955–959.
